# Uncovering shape signatures of resting‐state functional connectivity by geometric deep learning on Riemannian manifold

**DOI:** 10.1002/hbm.25897

**Published:** 2022-05-10

**Authors:** Tingting Dan, Zhuobin Huang, Hongmin Cai, Robert G. Lyday, Paul J. Laurienti, Guorong Wu

**Affiliations:** ^1^ School of Computer Science and Engineering South China University of Technology Guangzhou China; ^2^ Department of Radiology Wake Forest School of Medicine Winston Salem North Carolina USA; ^3^ Department of Psychiatry University of North Carolina at Chapel Hill Chapel Hill North Carolina USA; ^4^ Department of Computer Science University of North Carolina at Chapel Hill Chapel Hill North Carolina USA; ^5^ Department of Statistics and Operations Research University of North Carolina at Chapel Hill Chapel Hill North Carolina USA; ^6^ Carolina Institute for Developmental Disabilities (CIDD) University of North Carolina at Chapel Hill Chapel Hill North Carolina USA; ^7^ UNC NeuroScience Center University of North Carolina at Chapel Hill Chapel Hill North Carolina USA

**Keywords:** deep learning, functional brain network, functional dynamics, Riemannian geometry, symmetric positive definite matrix

## Abstract

Functional neural activities manifest geometric patterns, as evidenced by the evolving network topology of functional connectivities (FC) even in the resting state. In this work, we propose a novel manifold‐based geometric neural network for functional brain networks (called “Geo‐Net4Net” for short) to learn the intrinsic low‐dimensional feature representations of resting‐state brain networks on the Riemannian manifold. This tool allows us to answer the scientific question of how the spontaneous fluctuation of FC supports behavior and cognition. We deploy a set of positive maps and rectified linear unit (ReLU) layers to uncover the intrinsic low‐dimensional feature representations of functional brain networks on the Riemannian manifold taking advantage of the symmetric positive‐definite (SPD) form of the correlation matrices. Due to the lack of well‐defined ground truth in the resting state, existing learning‐based methods are limited to unsupervised methodologies. To go beyond this boundary, we propose to self‐supervise the feature representation learning of resting‐state functional networks by leveraging the task‐based counterparts occurring before and after the underlying resting state. With this extra heuristic, our Geo‐Net4Net allows us to establish a more reasonable understanding of resting‐state FCs by capturing the geometric patterns (aka. spectral/shape signature) associated with resting states on the Riemannian manifold. We have conducted extensive experiments on both simulated data and task‐based functional resonance magnetic imaging (fMRI) data from the Human Connectome Project (HCP) database, where our Geo‐Net4Net not only achieves more accurate change detection results than other state‐of‐the‐art counterpart methods but also yields ubiquitous geometric patterns that manifest putative insights into brain function.

## Graphical abstract

- Learning low‐dimensional feature signatures of resting‐state brain network on Riemannian manifold.
- Capturing the geometric patterns manifested in evolving functional fluctuations in resting state.
- Answering the question of how the spontaneous fluctuation of FC supports behavior and cognition.

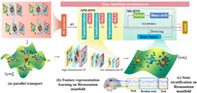

## 1 INTRODUCTION

One of the fundamental scientific problems in neuroscience is to have a good understanding of how cognition and behavior emerge from brain function. In the last couple of decades, striking efforts have been made to establish structural and functional brain mapping in vivo using cutting‐edge neuroimaging technologies. For instance, functional resonance magnetic imaging (fMRI) technology has been widely used to characterize the synchronized functional fluctuations between spatially distinct brain regions by examining the temporal correlation of blood‐oxygen‐level‐dependent (BOLD) signals (Buckner et al., 2013).

The major body of current functional studies strives to understand the brain networks generated using functional connectivity (FC) underlying normal and abnormal brain function. Due to the high dimensionality of whole‐brain networks, dimensionality reduction techniques are often used to study the connectivity characteristics using compact and intrinsic feature representations. It is common to use graph theory to generate a set of network measurements, such as connectivity degree and local clustering coefficient (Rubinov & Sporns, 2010) to characterize the FC profile for each node and the entire functional brain network. Although the feature dimensionality is significantly reduced using these measurements, the system‐level information of whole‐brain connectivity can be overly simplified. Data‐driven approaches such as principal component analysis (PCA) and t‐distributed stochastic neighbor embedding (t‐SNE) have also been widely used to capture the brain network variation across individuals or task conditions (Bahrami et al., 2019; Billings et al., 2017). Similar to the applications in computer vision and machine learning, it is a common practice to vectorize the whole functional brain network into a data array. By doing so, the non‐Euclidean topological structure of the functional network is distorted, which could result in limited insights or even inaccurate conclusions from the low‐dimensional network representations.

Since Pearson's correlations are often used to measure the strength of FC between two nodes in a brain network (Amaral et al., 2008; Heuvel & Pol, 2010), each functional brain network can be quantified by a symmetric positive‐definite (SPD) matrix, which allows for the assessment of brain networks on well‐studied Riemannian manifolds. To facilitate the learning and explanation, the FC matrix needs to be mapped from the Riemannian space to the Euclidean space by projecting it onto a tangent plane of the Riemannian manifold and vice versa. In existing work, ( Dai et al., 2019) proposed to align the FC matric from different subjects within Riemannian manifold, which removes the differences between multiple sessions of a single subject (Yair et al., 2019). Since all the algebraic operations on functional brain networks are performed on the Riemannian manifold of SPD matrices, the network topology is well maintained during network inference. Thus, manifold‐based methods often achieve more accurate network analysis results than the counterpart approaches using Euclidean operations. For instance, a novel dimensionality reduction method is proposed by Dai, Zhang, and Srivastava (2020), using the SPD matrix on the Riemannian manifold. The authors demonstrated the improved classification accuracy of functional tasks based on the low‐dimensional FC matrices, compared with the conventional approaches using PCA to reduce the dimensionality.

Recently, the research focus of fMRI studies has been shifted to functional dynamics since mounting evidence shows the brain function fluctuates even in the resting state (Filippi et al., 2019). In addition, it has been frequently reported that the dynamic behavior of functional brain networks is closely associated with the development of neurological diseases. In light of this, numerous methods have been proposed to characterize functional dynamics from the observed BOLD signals. For example, the Bayesian‐based statistical inference has been used to partition the time course into segments by modeling the statistics of BOLD signals and the temporal transition probability (Cribben et al., 2012; Xu & Lindquist, 2015). A recurrent neural network (RNN) has been proposed in the work of Li and Fan (2018) by considering the brain state change detection as a classification problem where the RNN is trained to predict the task pseudo‐label based on the FC signatures vectors generated through non‐negative matrix decomposition (Li et al., 2017). More recently, an increasing number of works on analyzing dynamic FC for boosting classification/recognition task of brain state have been introduced. Such as, a locally linear embedding of dynamic FC has been introduced by Yang et al. (2019), which recognizes the different FC states by detecting the differences between brain state groups. To further consider the spatio‐temporal variations that define dynamic brain states during task performance, Chan et al. (2020) proposed a customized salient pattern over time and space (SPOTS) to classify brain states. Adopted a deep network model that considers the subtle time‐varying patterns in dynamic FC for capturing temporal and spatial features of FC sequences simultaneously. Gao et al. (2021) presented a dynamic FC embedding, which can preserve proper temporal progression among brain states.

Mounting evidence shows that the human brain is a network with distributed processing ability (Sporns, 2011; Watts & Strogatz, 1998), where the cognitive task information is transferred between brain regions through network topology (Ito et al., 2017). Following this lead, it is important to understand the geometry of functional brain networks and further characterize the dynamic behavior of brain state change on top of the evolving geometric patterns. To that end, we propose a manifold‐based deep learning approach to uncover the dynamic brain mapping of resting‐state FC on the Riemannian manifold.

Specifically, we regard each functional brain network as an instance of SPD matrix on the Riemannian manifold. To capture the functional dynamics, we use the sliding window technique (Hutchison et al., 2013) to construct a set of functional brain networks, which constitute a functional time series of SPD matrices on the Riemannian manifold. Since we are interested in uncovering the intrinsic resting‐state FC map across individuals, we first remove the external intersubject variations from the functional brain networks by parallel transporting all SPD matrices to a reference location on the Riemannian manifold (Yair et al., 2019). Given the aligned trajectories of functional brain networks, we present a manifold‐based deep neural network (DNN) to learn the low‐dimensional feature representations for resting‐state FC using a set of positive maps and ReLU layers, which produces dimension reduced SPD matrices. To gain more heuristics into the learning process, we propose to include the task‐based FC matrices into the feature representation learning. Specifically, we schedule the task‐based fMRI scans before and after the resting state. Thus, we design a pretext task^1^ (Jing & Tian, 2020) that encourages the trajectory of the learned low‐dimensional SPD matrices to be aligned with the underlying change of brain states in each task‐resting alternating fMRI scan. More specifically, we cast this pretext task into a change point detection process by stratifying SPD matrices, which are achieved by a mean shift‐based recurrent neural network (MS‐RNN) on the Riemannian manifold. All the learning components are tailored to maintain and capture the geometric patterns on the Riemannian manifold. Since our DNN is designed to discover the geometric patterns of functional brain networks, we name our proposed network “Geo‐Net4Net” for short. It is worth noting that we use change point detection, instead of cognitive task recognition, for self‐supervising the pretext task for the following two reasons. (1) Recognizing/classifying cognitive tasks is not only much more challenging than change point detection but also difficult to scale up to a large number of tasks. (2) Recognizing each cognitive task is loosely correlated with functional dynamics. That is, recognizing each cognitive task does not necessarily lead to the accurate detection of brain state changes, which is the main focus of this work. Since it has been consistently reported in the literature that the shift of network topology is closely related to the cognitive tasks, characterizing the change in each SPD matrix sequence eventually promotes the temporal coherence of learned functional feature representations on the Riemannian manifold.

We demonstrate the performance of our Geo‐Net4Net in the following experiments. First, we validate the low‐dimensional feature representations through the accuracy of brain state change detection on both simulated data and working memory fMRI data from the HCP database. Compared to the current learning‐based change detection methods, our Geo‐Net4Net achieves not only more accurate detection results of brain state change but also higher replicability and scalability on the test–retest experiments. Second, we analyze the eigen‐spectrum of functional brain networks, which form the spectral pattern of FC for each cognitive task. Since the geometry of FC matrices is well‐preserved in the learning process, our Geo‐Net4Net offers a new window to understand the intrinsic FC maps of the resting state in the low‐dimensional Riemannian manifold of SPD matrix. Specifically, we explore the eigen‐spectrum of resting‐state networks and the associated functional circuits, as a first‐ever attempt in the neuroscience field, where we demonstrate the potential of identifying state‐based transitions and functional brain network dynamics in the resting brain using the Riemannian manifold.

## 2 METHODS

### 2.1 Geo‐Net4Net overview

**FIGURE 1:**
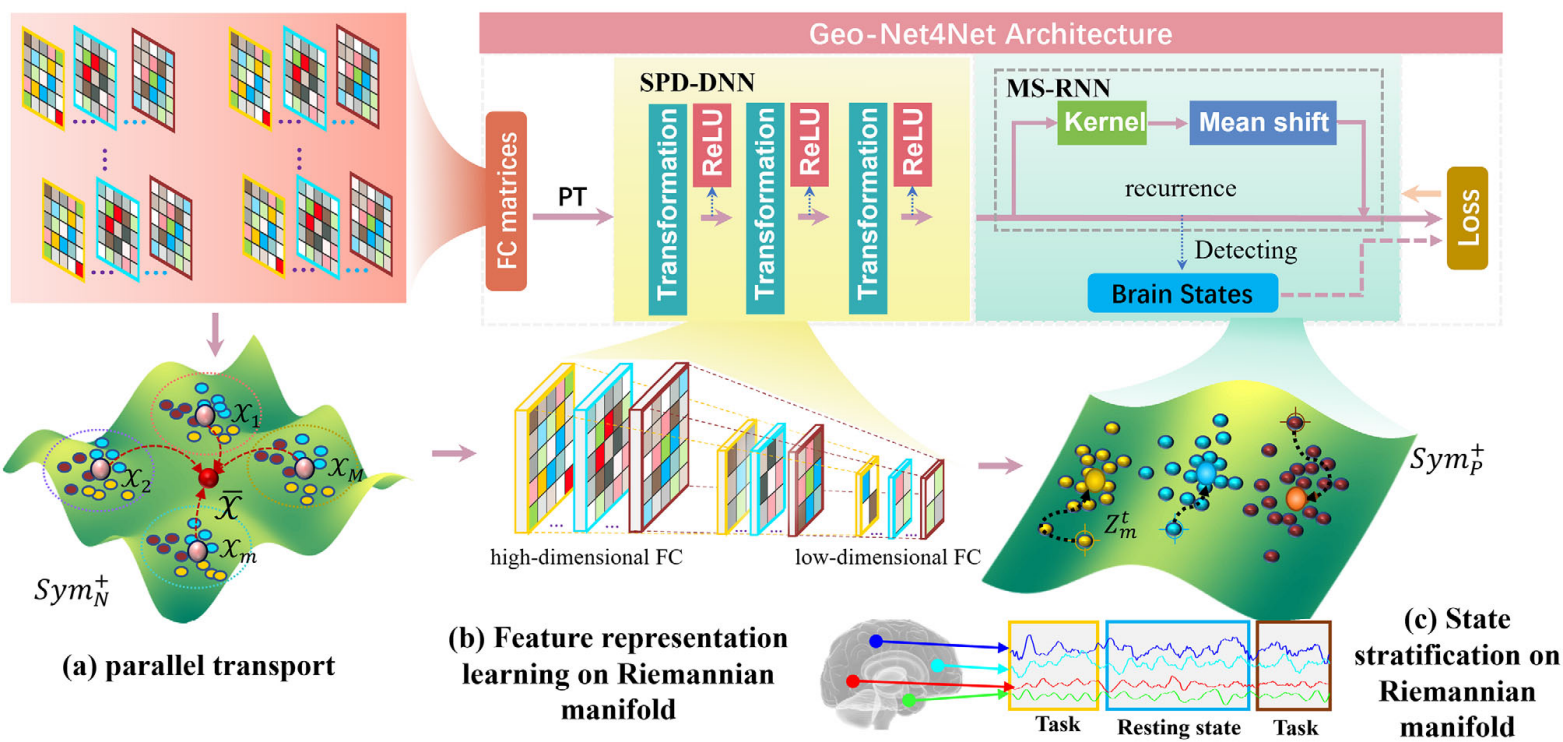
Overview of our self‐supervised learning framework for low‐dimensional feature representations for resting‐state functional connectivity (FC). The backbone is the manifold‐based deep neural network (Geo‐Net4Net), where the input is the aligned FC matrices on the Riemannian manifold using parallel transport technique (a). The feature‐learning component (b) consists of a set of symmetric positive‐definite (SPD) transformation and rectified linear unit (ReLU) layers. Furthermore, we use the task‐based FCs to self‐supervise the feature learning by requiring the learned low‐dimensional feature representations to reflect the change of brain states, which is implemented in an recurrent neural network (RNN) architecture (c).

The input to our neural network is a set of FC matrices constructed using the sliding window technique, where the whole sequence is alternated between cognitive tasks and resting states (see Section 3.1). The architecture overview of our Geo‐Net4Net is shown in Figure 1, which consists of three computational components. (1) Removing subject‐to‐subject variations by parallel transportation (PT). A large portion of network differences on Riemannian manifold can be attributed to the individual variations in the underlying brain structure/function. Hence, we first align each trajectory of FC matrices to a latent population mean on the Riemannian manifold, as a preprocessing step before training Geo‐Net4Net. By doing so, we remove the intersubject variations leaving the task‐specific variations. (2) Learning feature representations for FC. We present a SPD‐based deep neural network (SPD‐DNN) to learn the low‐dimensional FC feature representations, consisting of a set of SPD matrix dimensionality reduction (i.e., SPD transformation) and ReLU layers. (3) Self‐supervised learning of resting‐state FC through the detection of brain state changes. We leverage the transition between task and resting‐state to steer the feature representation learning of resting‐state FC by aligning the trajectory of learned FC feature representation with the functional tasks. The output of our Geo‐Net4Net is the low‐dimensional manifold feature representations for task‐based and resting‐state FC. A major methodological advance is that the method allows us to investigate network transitions in the resting‐state even in the absence of predefined transitions that exist when switching between cognitive tasks. We introduce the application of Geo‐Net4Net in functional brain network analysis in Section 2.5. Specifically, since the geometry of FC matrices is well maintained in the learning process, the geometric patterns constitute the spectral signature of each functional task.

### 2.2 Parallel transport of FC matrices on the Riemannian manifold

Suppose we have M fMRI scans in the training dataset. We parcellate each brain into N regions and then generate N mean time courses of BOLD signals, where each BOLD signal has T time points. Without the loss of generality, we assume that there are in total Q brain states $\Pi =\left\{\gamma_{q}|q=1,\ldots ,Q\right\}$ during each scan (including tasks and resting states). To capture the functional dynamics, we adopt the sliding window scheme (Allen et al., 2014) to construct a FC matrix trajectory $X_{m}=\{X_{m}^{t}\left|t=1,\ldots ,T\right\}\;\left(m=1,\ldots ,M\right)$ for $m^{\mathrm{th}}$ fMRI scan, where each $X_{m}^{t}$ is a N×N Pearson's correlation matrix of BOLD signals within the sliding window centered at scanning time t. In most fMRI studies, it is a common practice to assume the BOLD signal at each region is independent and their correlation matrix is a full‐rank matrix given the sufficient width of the sliding window. Thus, the resulting FC matrix is symmetric and positive‐definite (SPD) matrix, that is, $X_{m}^{t}\in \mathrm{Sy}m_{N}^{+}$.

Due to the subject‐to‐subject variation of brain structures, it is highly possible that there exist external differences of FC matrices across individuals, which might not be relevant to the state change detection. To remove the external subject‐to‐subject variations of FC, we use the parallel transporting (PT) technique (Yair et al., 2019) to align each trajectory of FC matrices to a reference center on the Riemannian manifold, as shown in Figure 1a. Thus, the remaining difference is supposed to be associated with the intrinsic representation for the underlying functional task. On the other hand, we consider functional dynamics as a trajectory of time (functional data) on the Riemannian manifold, since different trajectories might have different starting points and paces, parallel transport is necessary to align all trajectories on the Riemannian manifold. After that, the common patterns averaged in the population can be regarded as the intrinsic patterns that are only specific to the underlying brain functions. Following the domain adaptation algorithm in the study by Yair et al. (2019), we iteratively apply the following three major steps to align the entire sequence of FC matrices to the population center.

1. Estimate subject‐specific mean within each scan. We consider $X_{m}=\{X_{m}^{t}\left|t=1,\ldots ,T\right\}\;$ as functional data on the Riemannian manifold that evolves over time. Then we estimate the subject‐specific Fréchet mean of $X_{m}$ on Riemannian manifold (Yair et al., 2019), denoted by ${\bar{X}}_{m}$ (pink point in Figure 1a), which has the shortest geodesic distances to all FC matrices $X_{m}^{t}$. Please see the Appendix A for the detail of estimating ${\bar{X}}_{m}$ from $\{X_{m}^{t}\left|t=1,\ldots ,T\right\}$.
2. Estimate population‐wise mean across individuals. Given M subject‐specific mean ${\left\{{\bar{X}}_{m}\right\}}_{m=1}^{M}$, we estimate the population center $\bar{X}\;$(red point in Figure 1a) using the Fréchet mean estimation method in Appendix A.
3. Parallel transport individual SPD trajectory to the population center. Given the population center $\bar{X}$, we parallel transport each $X_{m}^{t}$ to $\bar{X}$ on the Riemannian manifold by $\Gamma_{X_{m}^{t}\to \bar{X}}=\mathrm{Es}E^{T}$, where $E={\left(\bar{X}{\left(X_{m}^{t}\right)}^{-\frac{1}{2}}\right)}^{\frac{1}{2}}$ and s is the tangent vector of $X_{m}^{t}$ at $\bar{X}$, that is, $s=\log_{\bar{X}}X_{m}^{t}$ (please see Appendix A for detail).

### 2.3 Feature representation learning for FC

To capture the intrinsic low‐dimensional feature representations for FC while preserving the geometrical information of network topology, we adapt a manifold‐based network (Huang & Gool, 2017) to learn the low‐dimensional geometric feature representation on the Riemannian manifold of SPD matrix. In a nutshell, the architecture of the manifold‐based network for SPD matrix learning is structured in a layer‐by‐layer manner as the popular DNN. The major difference between SPD‐DNN and regular DNN is that all operations in SPD‐DNN have been replaced by manifold algebra tailed for SPD matrix. In what follows, we formulate the SPD‐DNN as a function $F_{\Theta }\left(X\right)$ with the network parameters Θ for inferring the low‐dimensional geometric feature representation $V\in \mathrm{Sy}m_{P}^{+}\left(P\ll N\right)$ from the high‐dimensional input $X\in \mathrm{Sy}m_{N}^{+}$. Since we apply the same SPD‐DNN $F_{\Theta }$ to all $X_{m}^{t}$ one after another in the SPD matrix sequence $X_{m}$, we drop the variable t (for time point) and subscript m (for subject) in Section 2.3, for clarity.

As shown in Figure 1b, the nonlinear dimension reduction in SPD‐DNN is achieved by alternatively applying matrix transformation and ReLU activation. Suppose that the dimensionality of SPD matrix has been reduced from $N_{k-1}\times N_{k-1}\;$ to $N_{k}\times N_{k}$, which forms the input to the *k* ^th^ (k=1,…,K) layer. We first learn a bilinear mapping $W_{k}$ to further reduce the dimensionality of SPD matrix from $N_{k-1}\times N_{k-1}$ to $N_{k}\times N_{k}$ by:

(1) $$X_{k}=f_{b}\left(X_{k-1}\right)=W_{k}X_{k-1}W_{k}^{T},$$

where $W_{k}\in {\mathbb{R}}^{N_{k}\times N_{k-1}}$ ($N_{k}<N_{k-1}$). Note $f_{b}$ is a positive mapping function that has been well studied in quantum mechanics (see chapter 8 in Nielsen & Chaung, 2000). Similar to the ReLU layer in DNN, the nonlinearity is obtained by a hard‐thresholding smoothing process in the spectrum domain of the underlying $X_{k}$ by:

(2) $$X_{k}=f_{r}\left(X_{k}\right)=U_{k}\max\left(\mathrm{\varepsilon I},\Lambda_{k}\right)U_{k}^{T},$$

where $U_{k}$ and $\Lambda_{k}$ are eigenvectors and the diagonal matrix of corresponding eigenvalues. ε is a predefined scalar that controls the regularization of rectifying smaller eigenvalues. At the end of *K* ^th^ layer, the output is the low‐dimensional SPD matrix $V\in \mathrm{Sy}m_{P}^{+}\;\left(P=N_{K}\right)$, as shown in Figure 1b.

It is clear that the network hyper‐parameters Θ of SPD‐DNN consists of a set of mapping matrices, that is, $\Theta =\left\{W_{k}|k=1,\ldots ,K\right\}$, which project each sequence of FC matrices $X_{m}$ from $\mathrm{Sy}m_{N}^{+}$ manifold to a low‐dimensional SPD matrix sequence $V_{m}=\left\{V_{m}^{t}|t=1,\ldots ,T\right\}\;$ in $\mathrm{Sy}m_{P}^{+}$ (P≪N), it sets the stage to characterize the dynamic FC changes on the Riemannian manifold below.

### 2.4 Self‐supervised feature representation learning using task transition point

Due to the lack of the known characteristics of the resting state FC, it is difficult to cast the training of SPD‐DNN in a supervised manner. In this regard, we resort to task‐based FCs and use the prior knowledge of state changes to facilitate the feature representation learning of resting‐state FC. Although the detection of brain state changes is not directly related to learning the low‐dimensional FC feature maps on the Riemannian manifold, a good characterization of the FC is supposed to predict the transition between the task and resting‐states reliably. To that end, we design a novel pretext task that stratifies the learned low‐dimensional SPD matrices into the segments that are aligned with the timeline of the prescheduled cognitive tasks in each fMRI scan. We cast this pretext task as a change detection problem of identifying the distribution modes of the learned FC matrices, which is driven by a mean‐shift (MS) process on the Riemannian manifold. Specifically, we reckon that the evolution of brain states is reflected by the distribution of SPD matrices on the manifold, where the SPD matrices belonging to the same brain state should fall into the same cluster, while clusters are separable across brain states.

In general, MS operates in an iterative manner, which alternates two considerable steps (as shown in Figure 1c): (1) estimating the MS vector $Z_{m}^{t}$ for each instance $V_{m}^{t}$ and (2) updating the distribution of $V_{m}$. Specifically, we estimate the MS vector for each instance, pointing to the weighted average mass center of all other instances, where the weight is measured by the pairwise kernel distance. We use the function $\psi_{h}\left(V_{m}^{t},\sigma \right)$ to denote the calculation of MS vector $Z_{m}^{t}$, where σ is the kernel size. After that, it is straightforward to update each instance to the new location by following the direction of MS vectors, that is, $V_{m}^{t}\leftarrow \psi_{z}\left(V_{m}^{t},Z_{m}^{t}\right)$. Since the data instance is the SPD matrix on the Riemannian manifold, we define the functions $\psi_{h}$ and $\psi_{z}$ using Riemannian algebra (please see Appendix B).

To integrate the MS process with SPD‐DNN, we propose to encapsulate it in a recurrent neural network (denoted by MS‐RNN) with the kernel sizes σ as the trainable parameters in the MS‐RNN. Although we perform MS on the Riemannian manifold, the feed‐forward process in MS‐RNN also consists of a set of matrix operations. As shown in Figure 1c, the input to MS‐RNN is a time course of the low‐dimensional geometric FC representations $V_{m}$ for each subject. Similar to the SPD‐DNN, we formulate MS‐RNN as a function $\Psi_{\phi }\left(V_{m}\right)=\psi_{z}\left(\psi_{h}\left(V_{m}\right)\right)$ with parameter $\phi =\left\{\sigma_{e}|e=1,\ldots ,E\right\}$, where we suppose MS‐RNN has E layers. The output is the stratified FC representations ${\hat{V}}_{m}=\Psi_{\phi }\left(V_{m}\right)$, which are supposed to collapse to several modes (cluster centers). After that, we group the obtained modes to produce a set of grouping results $\left\{y_{m}^{t}\left|t=1,\ldots ,T\right\}\right)$. Therefore, the grouped brain states can steer the learning of low‐dimensional geometric feature representation via minimizing the loss function in a self‐supervised manner. Given the brain state $y_{m}^{t}\in \Pi$ for each $V_{m}^{t}$, we expect the pair $V_{m}^{t}$ and $V_{m}^{t^{\prime }}$ ($t\neq t^{\prime }$) bears a small manifold distance $g_{m}^{tt^{\prime }}$ (i.e., high similarity $d_{m}^{tt^{\prime }}$) if they are in the same brain state, that is, $y_{m}^{t}=y_{m}^{t^{\prime }}$. Otherwise ($y_{m}^{t}\neq y_{m}^{t^{\prime }}$), their similarity $d_{m}^{tt^{\prime }}$ between $V_{m}^{t}$ and $V_{m}^{t^{\prime }}$ should be as small as possible, where $g_{m}^{tt^{\prime }}={\left\|\log\left({\hat{V}}_{m}^{t}\right)-\log\left({\hat{V}}_{m}^{t^{\prime }}\right)\right\|}_{F}$ is defined as the geodesic distance between SPD matrices $V_{m}^{t}$ and $V_{m}^{t^{\prime }}$, the pairwise similarity matrix is derived by:

(3) $$d_{m}^{tt^{\prime }}=\frac{1}{1+g_{m}^{tt^{\prime }}}.$$

Therefore, the loss function ℓ of our Geo‐Net4Net is given by:

(4) $$\ell =\sum_{m=1}^{M}\sum_{t,t^{\prime }=1}^{T}\left(1_{\left\{y_{m}^{t}=y_{m}^{t^{\prime }}\right\}}\left({1-d}_{m}^{\mathrm{tt}\prime }\right)+1_{\left\{y_{m}^{t}\neq y_{m}^{t^{\prime }}\right\}}{\left[d_{m}^{\mathrm{tt}\prime }-\alpha \right]}_{+}\right)$$

where the scalar α controls the maximum margin for negative pairs of time points that bear with different functional tasks.

We learn the functional FC representations by finding the best network parameters Θ and ϕ that minimize the loss function ℓ. Given the input $X_{m}$, the output to our Geo‐Net4Net is ${\hat{V}}_{m}=\Psi_{\phi }\left(F_{\Theta }\left(X_{m}\right)\right)$. Specifically, the function of SPD‐DNN can be formulated as $F=\underset{K}{\underbrace{\left(f_{b}\circ f_{r}\right)\circ \ldots \circ \left(f_{b}\circ f_{r}\right)}}$. Likewise, the function of MS‐RNN is boiled down to $\Psi =\underset{E}{\underbrace{\left(\psi_{h}\circ \psi_{z}\right)\circ \ldots \circ \left(\psi_{h}\circ \psi_{z}\right)}}$. We show the detail of tuning network parameters $\left\{\Theta ,\phi \right\}$ in the back‐propagation process in Appendix C.

### 2.5 Application of Geo‐Net4Net to resting‐state functional networks

Since the geometry of FC matrices is well maintained in the whole learning process, we conceptualize that the geometric patterns constitute the spectral signature of the resting state FC. Specifically, we consider each instance of FC $X_{m}^{t}$ is governed by the eigen‐system of its low‐dimensional FC footprint $V_{m}^{t}$ (output of SPD‐DNN) on the Riemannian manifold. In analogy to the shape analysis in computer vision (Cootes et al., 1995), the eigenvalues and eigenvectors of the population‐mean $\bar{V}$ (see Appendix A for the calculation of SPD matrix mean $\bar{V}$) yield the spectral bases of resting‐state FC on a population level. Then we can extrapolate the complete spectrum of resting‐state FCs by applying perturbations on these eigenvalues, which has the potential application in simulating ground‐truth data of resting‐state FC. Since these spectral bases are orthogonal and reflect the underlying network geometry, we can introduce the well‐studied physics concept such as power and energy into the network neuroscience, which allows us to understand the working mechanism of the entire resting‐state functional network with a greater mathematics insight. Furthermore, it is straightforward to reconstruct the high‐dimensional functional brain network from the low‐dimensional FC map $V_{m}^{t}$ by inversing the positive maps $f_{b}$ (Equation 1). Thus, our Geo‐Net4Net provides a new window to identify the focal network patterns with the underlying spectral bases.

## 3 EXPERIMENTAL RESULTS

In this section, we evaluated the performance of our Geo‐Net4Net on both simulated data and real task‐based fMRI data from the Human Connectome Project (HCP) (Barch et al., 2013), see the Section 3.1. Since Geo‐Net4Net is driven by the transition between brain states, we first evaluate the change detection accuracy in Section 3.2. We compared our Geo‐Net4Net with three methods, including (1) the spectral clustering method (SC) (Ng et al., 2001), (2) the recent clustering method by seeking for density peaks (DP) of the data distribution (Rodriguez & Laio, 2014), and (3) the graph‐based change detection method (dGE) (Lin et al., 2020). In contrast to our method, the counterpart methods first vectorize the FC matrices of the brain network and then perform change detection. The parameters of the counterpart methods use their default settings of published versions. We used the purity score (Dan et al., 2022; Huang, Cai, et al., 2021; Huang, Dan, et al., 2021) between the ground truth (predefined task schedules) and stratified brain states to evaluate the detection accuracy, which has been widely used in the computer vision area for assessing clustering accuracy. In Section 3.3, we demonstrated the spectral characteristics of geometric patterns from resting‐state functional networks by our Geo‐Net4Net, which is the first exploration in the neuroimaging field. Regarding the spec of PC, we use an Intel (R) Core (TM) i7‐8700 CPU @ 3.20 GHz PC without graphic card. For our Geo‐Net4Net, the learning rate is set as 0.01, W is initialized as a random semiorthogonal matrix, the rectifying threshold ε is set to $10^{-4}$, the kernel size of mean shift σ is initialized as 0.5.

### 3.1 Dataset description

#### 3.1.1 Simulated data

**FIGURE 2:**
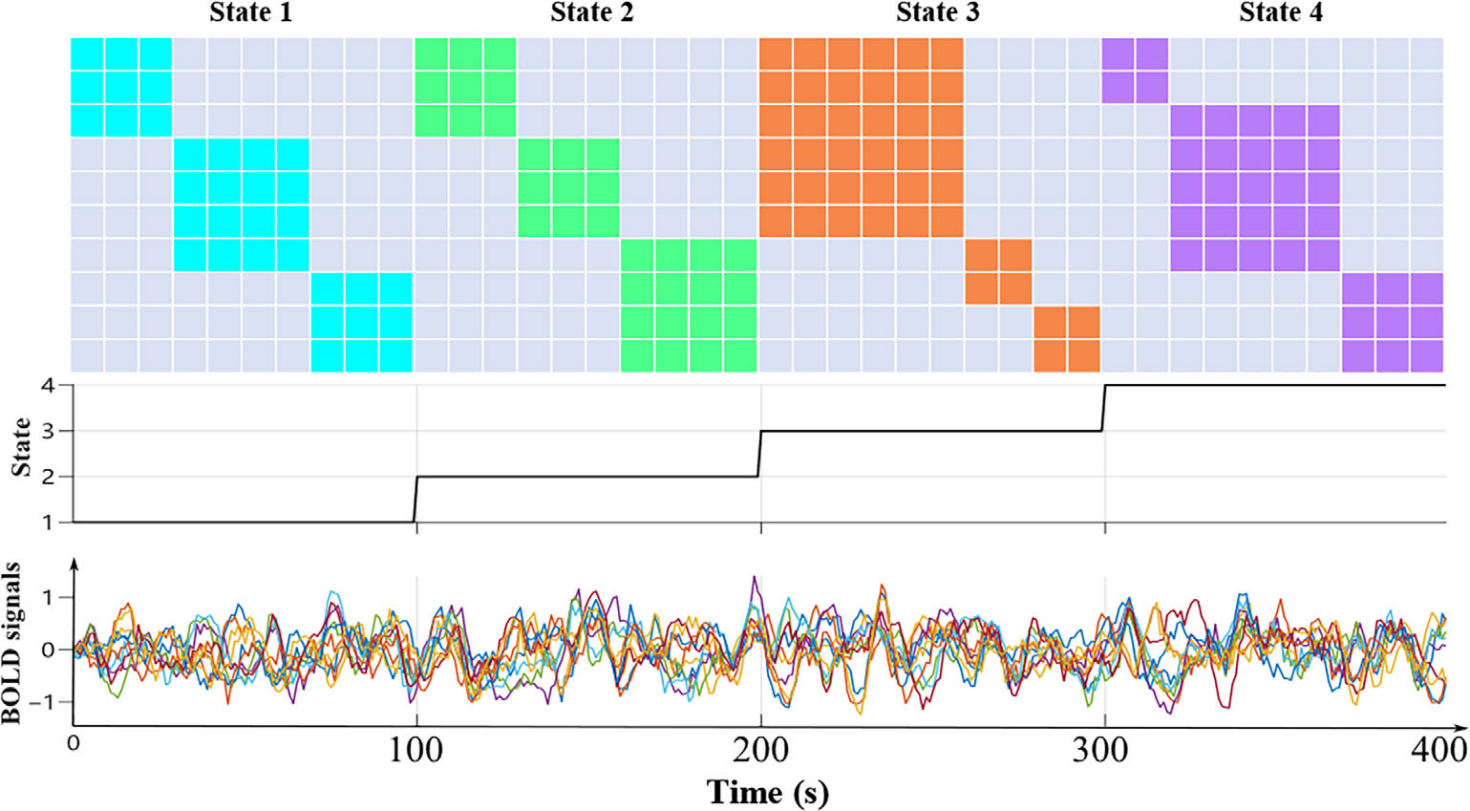
An example of simulated data with 10 brain regions and 4 brain states

The SimTB toolbox (Erhardt et al., 2012) was utilized to generate the simulated fMRI time series with four brain states (Q=4). Figure 2 (top) demonstrates four FC matrices for the predefined states, where each FC matrix consists of three modules (communities) along the diagonal line (State 1 in blue, State 2 in green, State 3 in orange, and State 4 in purple). For each possible pair of nodes with the same module, the degree of connectivity is set to one. Otherwise, it is assigned a value of zero concerning cross‐module connectivity. A total of 2000 sets of time series were simulated to evaluate the sensitivity of Geo‐Net4Net regarding window size and network size (number of nodes). Each simulation included the three change points (t=100,t=200,t=300) to separate the four states (shown in the middle of Figure 2) and each state lasted 100 s (shown in the bottom of Figure 2). The simulated time series were used to generate dynamic networks by the sliding window technology (Allen et al., 2014). The 2000 simulated network time series were separated into 900 training sets, 100 validation sets, and 1000 testing sets. We generated the simulated dynamic fMRI data with 10 regions for analyzing the *sensitivity* using different window sizes and increased the number of nodes to further evaluate the *scalability* in network sizes, specified in Section 3.2.1. For the scalability analyses, a window size of 25 time points was used to generate the networks.

#### 3.1.2 HCP human fMRI data

A total of 960 subjects were selected from the HCP database (Barch et al., 2013), each with test and retest fMRI scans of the working memory task. We split the whole fMRI data into a training set (425 subjects), validation set (55 subjects), and testing set (480 subjects), respectively. The task included 2‐back and 0‐back task conditions for body, place, face, and tool stimuli, as well as fixation periods. Note that there was a resting‐state period after two sequential cognitive task periods in an alternating fashion. We utilized the HCP minimally processed data that included distortion correction and had been warped to standard space (Glasser et al., 2013). Each fMRI scan consisted of 393 scanning time points. We used two scans for each subject, the left‐to‐right (LR) and right‐to‐left (RL), with one being used for test and the other for retest analyses. For each scan, ICA‐AROMA (Pruim, Mennes, Buitelaar, & Beckmann, 2015; Pruim, Mennes, van Rooij, et al., 2015) was used to remove motion signal artifacts based on temporal and spatial features in the data related to head motion. A band‐pass filter (0.009–0.08 Hz) was then applied to each scan and a regression was performed using mean tissue signals (GM, WM, and CSF), the six movement parameters and derivatives. The brain was parcellated into 268 brain regions using the Shen functional atlas (Shen et al., 2013) and the residual fMRI signal from all voxels in each parcel was averaged. The functional brain networks were then created by performing a cross‐correlation between each and every pair of network nodes (brain regions). Some analyses were limited to specific intrinsic subnetworks and subnetworks were used to help with results interpretations. We used seven intrinsic subnetworks commonly identified in functional brain networks: central executive network (CEN), visual network (VN), sensorimotor network (SMN), default mode network (DMN), dorsal attention network (DAN), salience network (SN), and the basal ganglia network (BGN). In addition, regions included in an unassigned (UA) group were located in areas with high signal dropout due to tissue/air artifacts near the paranasal sinuses. These subnetworks were identified using modularity analyses (Girvan & Newman, 2002; Moussa et al., 2012; Newman & Girvan, 2004) performed on functional brain networks collected in 22 normal young adults from a prior study (Mayhugh et al., 2016).

We evaluated (1) the *accuracy* of change detection in different network dimensions and window sizes, the *replicability* by applying the Geo‐Net4Net trained on test data to retest data and vice versa, the *necessity* of two proposed learning components (SPD‐DNN and MS‐RNN), described in Section 3.2.2, (2) the network spectrum of specific‐task FC brain mappings, followed by exploring the resting‐state FC eigen‐system spectrum (Section 3.3).

### 3.2 Evaluation of change detection using the learned low‐dimensional feature representations

#### 3.2.1 Change detection on simulated data

##### Sensitivity analysis on different window size

**FIGURE 3:**
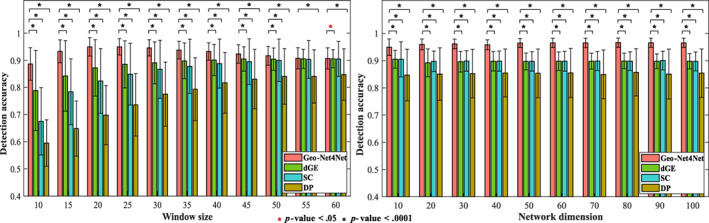
Change detection accuracy in simulated data. Left: The accuracy of state change detection for different window sizes for each of the four methods (note that a network size of 10×10 was used). Right: The accuracy of state change detection for different network sizes (note that a window size of 25 time points was used). The proposed Geo‐Net4Net significantly outperformed the other three comparison methods for most window sizes and all network dimensions. “*” denotes the significant improvement in *t*‐test (red: p<.05, black: p<.0001).

We extensively evaluate the accuracy of change detection on the simulated data with varied window sizes from 10 to 60 (time points). The left panel of Figure 3 shows the detection accuracy in terms of purity score by our Geo‐Net4Net (in red), dGE (in green), SC (in blue), and DP (in brown). Our proposed Geo‐Net4Net uniformly outperforms the other three counterpart methods w.r.t. all settings of window sizes, where “*” denotes significant improvement in paired *t*‐test.

##### Scalability regarding network size

We further estimate the detection accuracy as we increase network size from 10×10 to 100×100. The right panel of Figure 3 shows the detection accuracy in terms of purity score by our Geo‐Net4Net (in red), dGE (in green) SC (in blue), and DP (in brown). Again, our proposed our Geo‐Net4Net achieves the highest detection accuracy over the other three competing methods in all settings of network sizes, indicating that our Geo‐Net4Net is consistently robust in terms of network dimension.

#### 3.2.2 Change detection in human fMRI data

##### Evaluation accuracy across the large‐scale functional networks

**FIGURE 4:**
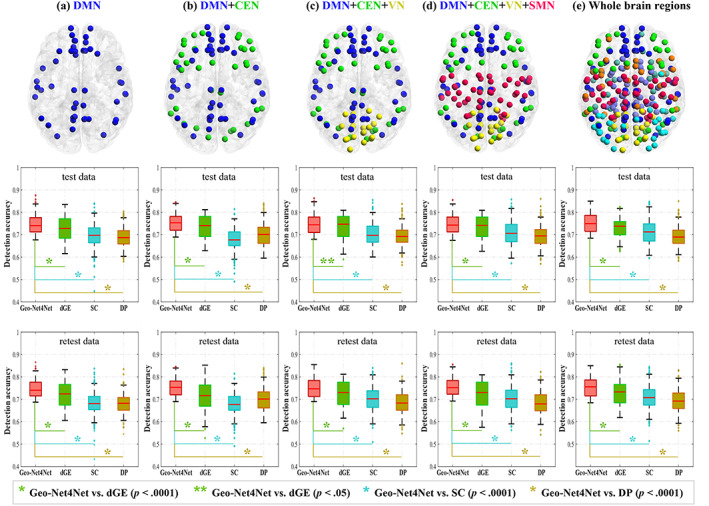
The detection accuracy for different network dimensions as we progressively include default mode network (DMN) (a), central executive network (CEN) (b), visual network (VN) (c), sensorimotor network (SMN) (d), and remaining nodes to cover the whole brain (e).

We evaluated the accuracy of change detection methods across different network sizes. We increased the size of the networks by successively adding subnetworks to the DMN in the following order: CEN, VN, SMN, and finally all network nodes. For each scenario, we first mixed the training sets of the test/retest fMRI data to evaluate the accuracy of change detection. We then trained a deep model from the mixed training sets, including 850 series (425 participants × 2, test and retest scans) and then validated on 110 time series (55 participants × 2) in test and retest data, finally tested on the remaining 480 test fMRI data and the corresponding 480 retest fMRI data, respectively. The nodes included at the step in the analysis are illustrated at the top of Figure 4, and the corresponding detection results conducted on test and retest data are shown in Figure 4 (middle and bottom). With the expansion of the network size, the detection accuracy of the comparison methods swelled and subsided, the corresponding costs were also increased. In contrast, our method remained basically stable and the highest accuracy was yielded in the second combination (DMN + CEN). It is clear that Geo‐Net4Net consistently significant (p<.05) outperforms all the comparison methods on all network sizes, as highlighted by the ‘*’. As a result, the following change detection analyses were limited in the DMN + CEN brain regions.

##### Detection accuracy across window sizes in DMN + CEN

**FIGURE 5:**
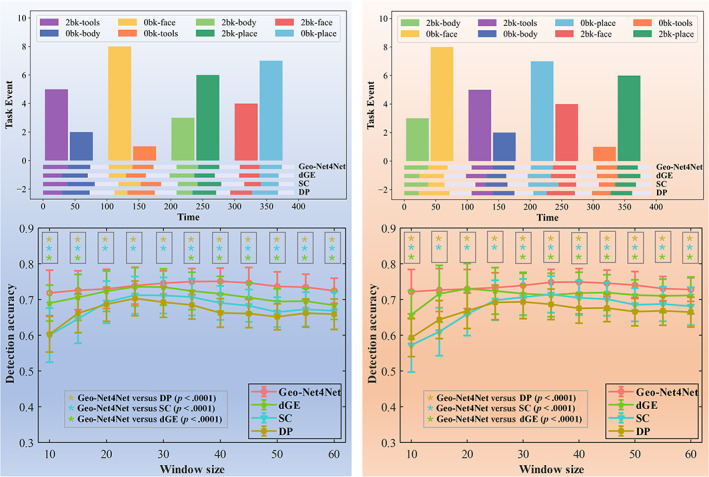
Top: Illustrations of change detection results by the Geo‐Net4Net, dGE, SC, and DP methods on test (in blue shadow) and retest data (in orange shadow). Bottom: The detection accuracies of change detection by Geo‐Net4Net (in red), dGE (in green), SC (in blue), and DP (in brown)

We trained models of different window sizes under the same data distribution as the previous experiment. Figure 5 (top) shows the change detection results of a representative participant for the test (Figure 5 left) and retest data (Figure 5 right). The bar plots with different colors and heights denote the predefined time schedule of functional tasks. The automatic detection results by Geo‐Net4Net, dGE, SC, and DP are displayed at the bottom of the bar plot with the color matched to the ground truth. By visually examining the temporal alignment between the predefined functional tasks and the automatic detection results, it is quite clear that Geo‐Net4Net yields more accurate predictions than the other three methods. Furthermore, we evaluated the accuracy of brain state change detection using different sliding window sizes, ranging from 10 to 60 (time points). The mean and standard deviation of purity scores are shown in Figure 5 (bottom) for test and retest data separately. Similar to the result of simulated data in Figure 3, our Geo‐Net4Net (in red) significantly outperformed (p<.0001) dGE (in green), SC (in blue), and DP (in brown) for most window sizes. Based on these results, we set the sliding window size to 40 time points in the following experiments.

##### Evaluation on replicability

We evaluated the replicability of the brain state change detections between test and retest data where the tasks were performed in a different order. Specifically, we first trained one Geo‐Net4Net using the test fMRI data solely (called Geo‐Net4Net‐LR) and another Geo‐Net4Net using the retest data only (called Geo‐Net4Net‐RL). Since each test and retest data set are paired, we split them into 425 as the training set, 55 validation set, and the remaining 480 as the application set in the same manner. Next, we applied the trained Geo‐Net4Net‐LR to not only the application sets of the test fMRI data but also the application set of the retest fMRI data where Geo‐Net4Net‐LR has not seen any instance of retest fMRI data in the training stage and vice versa. We evaluated such test/retest replicability and the accuracy of change detection on data with the same task order versus training/testing on data with different task orders, as summarized in Table 1. Statistically, there were no significant differences between the purity scores of two application scenarios. The finding implies that Geo‐Net4Net is highly replicability even when the task order is different between test and retest data.

**TABLE 1:** The detection results for evaluating the replicability of change detection

		Same task schedule versus different task schedule
Geo‐Net4Net‐LR	Mean	0.7508 versus 0.7474
	STD	0.0369 versus 0.0383
	*t‐*test	No significant difference found (p<.2608)
Geo‐Net4Net‐RL	Mean	0.7490 versus 0.7466
	STD	0.0374 versus 0.0405
	*t‐*test	No significant difference found (p<.4509)

##### Evaluation necessity of the proposed components for Geo‐Net4Net

To assess the necessity of the proposed two‐stage architecture of Geo‐Net4Net (see Figure 1), we conducted an ablation study on human fMRI data testing the change detection accuracy after turning on/off SPD‐DNN and/or MS‐RNN. With the SPD‐DNN stage turned off, the input to MS‐RNN is the time series of FC matrices generated using the sliding window procedure. By turning off the MS‐RNN, the learned geometric FC representations are used for classic spectral clustering. By shutting down all network components, the FC matrices are used to stratify the time points by spectrum clustering. Table 2 shows that each component plays an essential role in change detection, as evidenced by the significant difference (p<.0001) after turning off either or both components.

**TABLE 2:** Evaluation necessity of the SPD‐DNN and MS‐RNN in our Geo‐Net4Net

SPD‐DNN		×	×	√	√
MS‐RNN		×	√	×	√
Purity	Mean	0.693	0.712	0.729	**0.751**
	STD	0.057	0.053	0.059	**0.038**
*t‐*test		$p<10^{-4}$	$p<10^{-4}$	$p<10^{-4}$	Not applicable

*Note*: Mean and STD denote the mean and standard deviation of purity for the testing set. ‘×’ and ‘√’ indicate the absence and presence of the underlying component stage in the analysis, respectively. The bold values denote the best results, which have no statistical significance.

Abbreviations: DNN, deep neural network; MS, mean‐shift; RNN, recurrent neural network; SPD, symmetric and positive‐definite.

### 3.3 Spectral characteristics of FC brain mappings

#### 3.3.1 Exploration on the FC eigen‐spectrum

**FIGURE 6:**
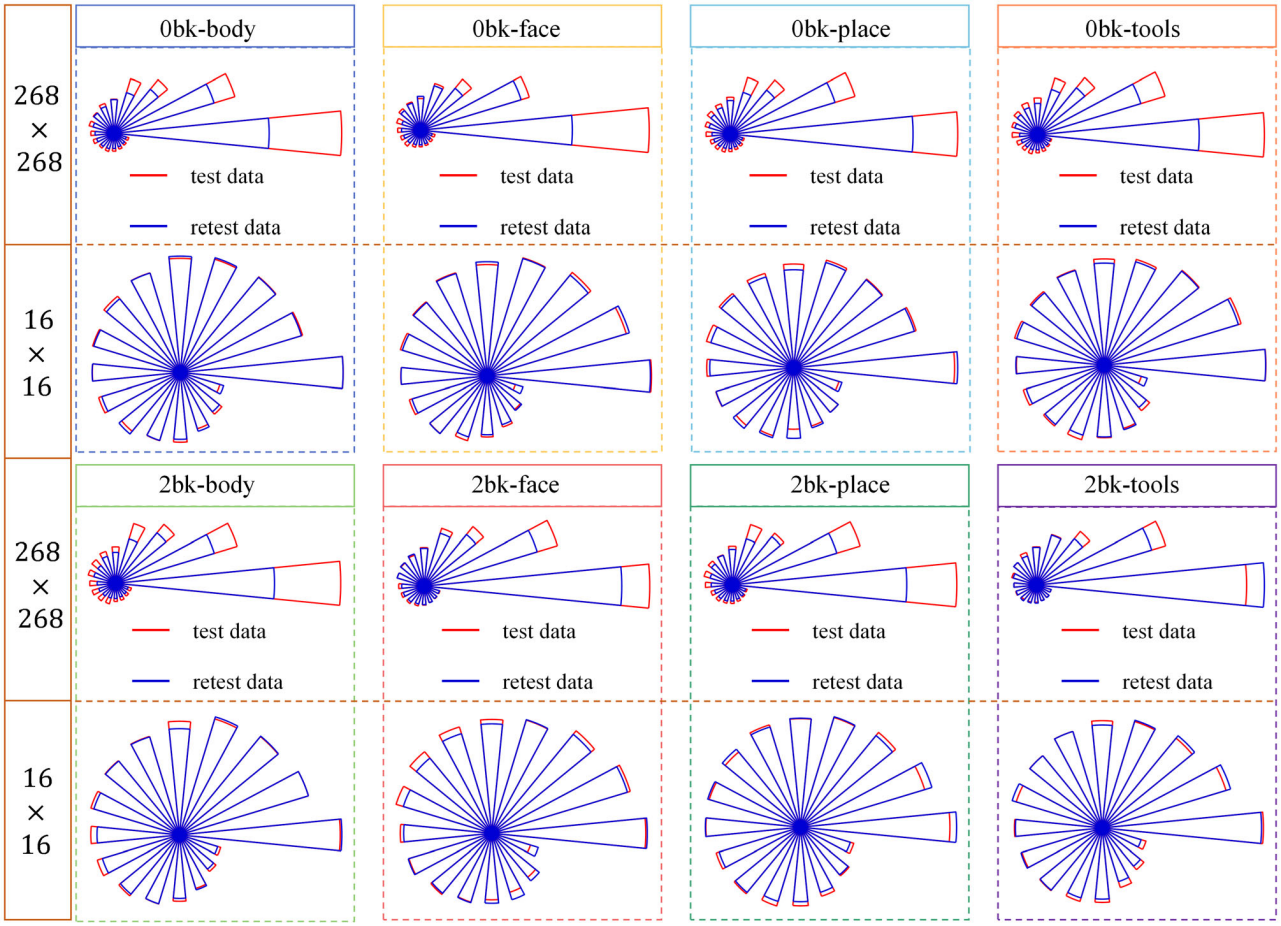
The consistency of eigen‐spectra between test (red plot) and retest (blue plot) data based on the average of original high‐dimensional functional brain networks (268×268) and the average of learned low‐dimensional FC feature representations $\left(16\times 16\right)$. For clarity, we only display the top 16 eigenvalues, where the degree of each eigenvalue is indicated by the radius. It is clear that the eigen‐spectra from the learned low‐dimensional feature representations exhibit better replicability in test/retest data across the functional tasks.

In this experiment, we chose the experimental result of the whole brain network (268 nodes) to analyze the spectral characteristics of FC brain mapping. To do so, we calculated the average SPD matrix (268×268) of the observed high‐dimensional function networks for each functional task and resting state. Similarly, we calculated the average low‐dimensional FC feature representations (16×16). Note that we used the method in Appendix A to estimate the mean on the Riemannian manifold rather than averaging the raw connectivity matrices. Then, we applied singular value decomposition (SVD) to the average SPD matrices. First, we evaluated the replicability of eigen‐spectra between test (red plot) and retest data (blue plot). We plotted the top 16 largest eigenvalues for each functional task in Figure 6, where we used the radius to reflect the degree of each eigenvalue. Each dashed box of Figure 6 is associated with a particular functional task and the eigen‐spectra for the high‐dimensional brain networks are on the top and the learned low‐dimensional feature representations on the bottom. It is clear that the eigen‐spectra from the output of Geo‐Net4Net are much more consistent (test vs. retest) than those derived from the original high‐dimensional functional networks.

**FIGURE 7:**
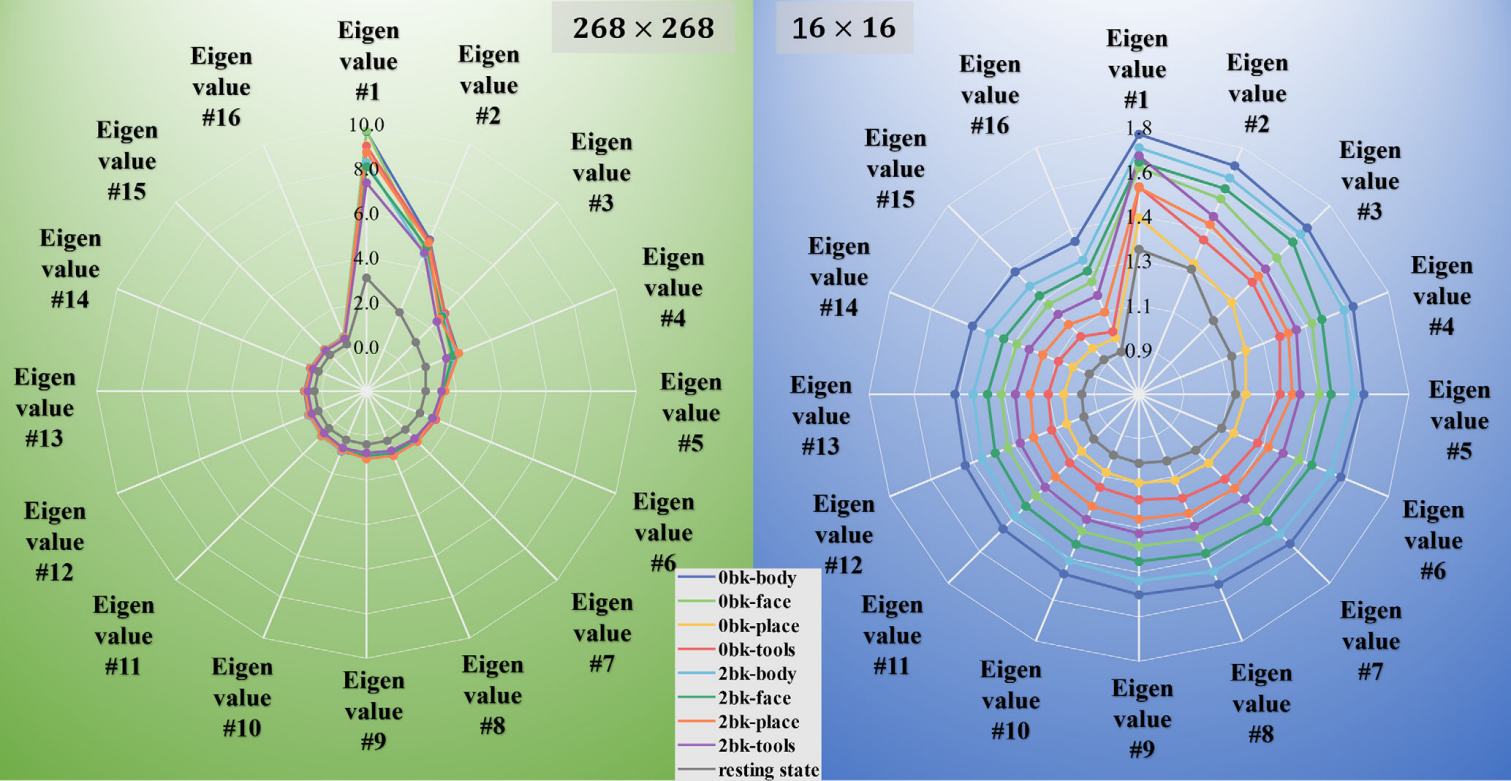
For each brain state (indicated by colors), we display the average eigenvalue trajectory from the eigen‐spectrum of the high‐dimensional FC matrices (left) and the learned low‐dimensional FC representations (right).

Second, we plotted the trajectory of eigenvalues (in decreasing order) for each brain state, including the resting state. Trajectories across brain states for the eigen‐spectra from 268×268 high‐dimensional FC matrices and 16×16 low‐dimensional feature representations are shown in the left and right panel of Figure 7, respectively. It is apparent that the learned low‐dimensional FC feature representations have more discriminative power for differentiating brain states in the spectral domain (the trajectories for different tasks are minimally overlapping) compared to the high‐dimensional data.

#### 3.3.2 Investigate the eigen‐spectrum energy between functional tasks and resting state

**FIGURE 8:**
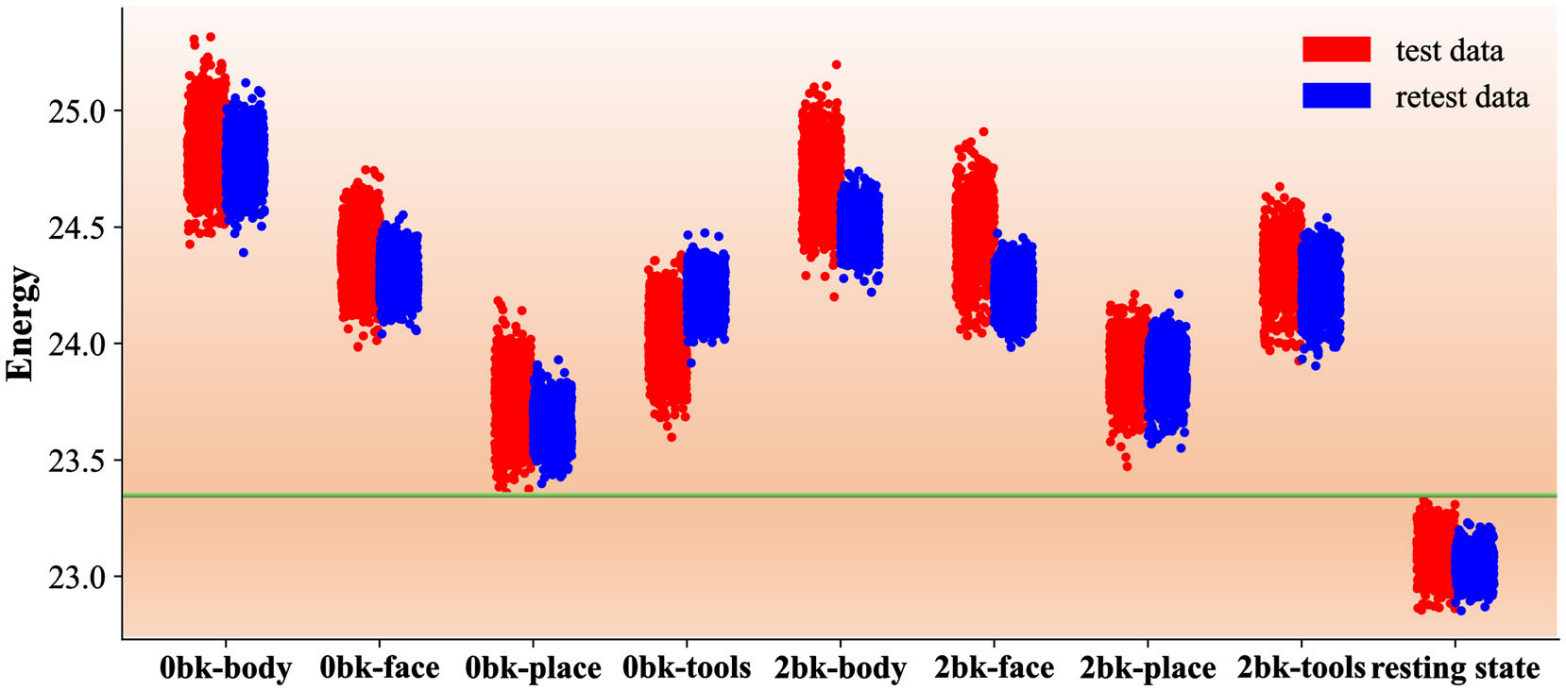
The Manhattan plot of energy of the learned low‐dimensional FC feature representations on test and retest data

In physics, the summation of squared eigenvalues indicates the energy of the underlying system. In light of this, we deploy a resampling test of system energy after we estimate the network parameters of our Geo‐Net4Net. Specifically, we withdraw 70% of samples of the learned low‐dimensional FC feature representations and calculate the eigenvalues for the underlying average FC features. We repeat this process 1000 times. Figure 8 shows the Manhattan plot of energy for each state, where red and blue denote the results using test and retest data, respectively. It is clear that the total energy of the brain system in the resting state (rightmost in Figure 8) is significantly lower than any of the brain states for the cognitive tasks. This intriguing finding motivates an examination of the characteristics of network circuits associated with eigen‐spectra in the resting state.

#### 3.3.3 Characteristics of eigen‐spectrum in the resting state

**FIGURE 9:**
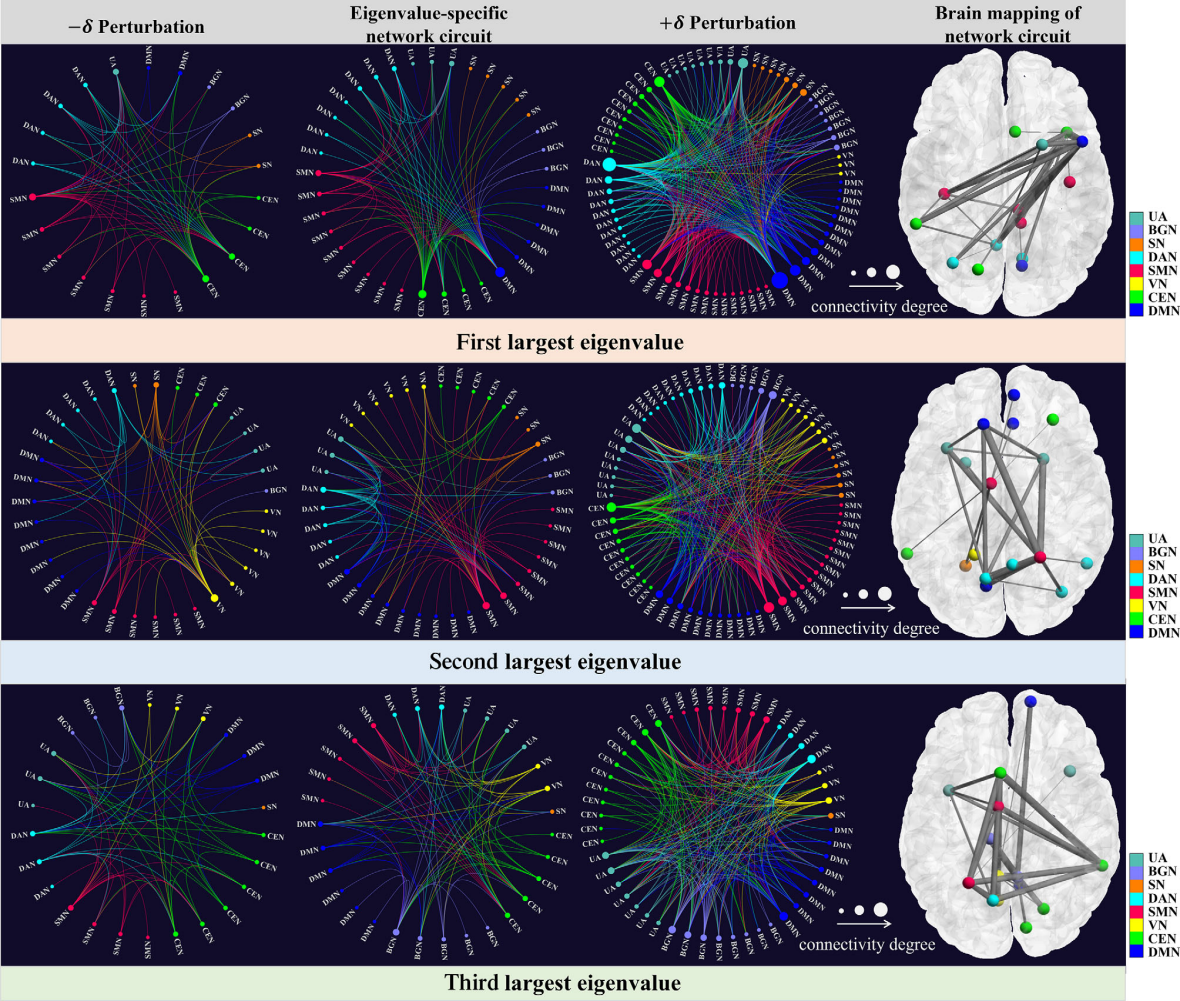
The eigenvalue‐specific network circuit (second column) and its variations are associated with –δ (first column) and +δ (third column) perturbations. The corresponding spatial maps of common network circuits in the brain are displayed in the last column. For clarity, we only display the top three eigenvectors (in each row).

Suppose we have the population average of low‐dimensional FC feature representations in the resting stage (please see Appendix A), that is, taking the mean of all resting‐state periods. Since the average is an SPD matrix, we can obtain a set of eigenvalues and the corresponding eigenvectors by SVD. Given the eigenvalues and eigenvectors, we can reconstruct the SPD matrix. We first extract each eigenvalue and corresponding eigenvector (in descending order) while nulling all other eigenvalues and eigenvectors. Next, we estimated the standard deviation in the population, denoted by δ (vector). Then we applied +δ and −δ perturbations on the underlying eigenvector and yielded the simulated low‐dimensional SPD matrices, which describe the range of variations driven by the eigenvalue under consideration. We ultimately reconstruct the eigenvalue‐specific network circuit based on the simulated low‐dimensional SPD matrix by inverting the positive maps $f_{b}$ (Equation 1). Note that we focus on the dimensionality reduction layer $f_{b}$ since we propose to verify the performance of the learned low‐dimensional FC representations, the remaining network layers are temporarily ignored. From the top to the bottom of Figure 9, we show the perturbation results for the top three eigenvalues, where the eigenvalue‐specific brain circuit is displayed in the second column and its variations of –δ and +δ perturbation are shown in the first and the third columns, respectively. The color of nodes and wirings represent the association with the large‐scale functional networks such as DMN and CEN. The size of each node reflects the connectivity degree. The DMN, SMN, and CEN were the most connected functional networks involved in the first, second, and third eigenvectors, respectively. The common network circuit (the connection shared across –δ and +δ perturbations) is mapped into the brain in the last column in Figure 9. As shown in Table 3, DMN, SMN, and CEN contribute a total of 55% of the total connectivities across eigenvalue‐specific network circuits, implying that these subnetworks are highly related to the working memory tasks. It is worth noting that the associations between these identified brain circuits and cognitive status in resting state have also been frequently reported in neuroscience literature (Chai et al., 2018; Dai, Zhang, Cai, et al., 2020; Hutchison et al., 2013).

**TABLE 3:** Connectivity degree of each subnetwork, –δ and +δ, respectively, denotes that the corresponding perturbations were applied, δ=0 has no perturbation.

		DMN (%)	SMN (%)	CEN (%)	VN (%)	DAN (%)	SN (%)	BGN (%)	UA (%)
First largest eigenvalue	–δ	6	30	30	0	15	4	4	10
	δ=0	**26**	20	22	0	16	4	3	9
	+δ	29	18	12	1	17	7	5	10
Second largest eigenvalue	–δ	17	22	16	7	10	5	9	14
	δ=0	18	**29**	7	7	16	6	4	14
	+δ	16	17	9	25	13	11	1	8
Third largest eigenvalue	–δ	18	12	15	7	12	2	17	16
	δ=0	15	13	**18**	10	13	2	18	12
	+δ	9	17	26	10	15	1	10	13
Total		**20**	**19**	**16**	6	14	5	9	12

*Note*: The bold values denote the maximum connectivity degrees, which have no statistical significance.

Abbreviations: BGN, basal ganglia network; CEN, central executive network; DAN, dorsal attention network; DMN, default mode network; SMN, sensorimotor network; SN, salience network; UA, unassigned; VN, visual network.

## 4 DISCUSSION

### 4.1 An in‐depth discussion on shape signature of FC in the resting stage

In our experiments, we demonstrated the performance of our manifold‐based DNN in detecting brain state changes and explored a new approach to investigate resting‐state FC using spectral analysis. The results shown in Figure 9 and Table 3 show the potential of our machine learning approach in understanding the functional dynamics, which bears the following three computation and neuroscience insights. First, we present a novel mathematical framework to understand functional dynamics by studying the system behavior. Specifically, we conceptualize that a functional brain network is the manifestation of an evolving system that vibrates as all other natural objects in the universe. Thus, each Eigensystem (Eigenvalues and Eigenvectors) is unique and underlines the functional task. Second, we introduce the classic PCA approach to characterize the statistics of system behaviors across individuals. We display three predominant components in Figure 9, which explain the largest three variations of functional dynamics in the spectrum domain. Third, we can further map the Eigenvectors to the brain and obtain the brain activation mapping of the regions that are associated with the underlying functional task, which offers a new window to understand the working mechanism of the human brain using data‐driven approaches.

It is worth noting that we used task‐fMRI to constrain the feature representation learning for the resting state. Also, the HCP data used in this work have a relatively shorter time period of the resting state than the tasks. Considering the nature of self‐supervised learning scenario, it is necessary to evaluate our method on the specifically designed fMRI sequence in future work.

### 4.2 Future work

The overarching goal of our method is to understand how the fluctuation of brain states results in diverse brain functions by characterizing the task‐specific geometric patterns. In this early stage work, the major contribution of this work is a collection of Riemannian algebra tailored for the manifold‐based deep model, which allows us to understand the intrinsic geometric patterns with great mathematics insight. We demonstrate the potential in detecting brain state change and reverse‐engineering the brain circuit (a collection of activated brain regions) associated with the underlying functional task. Our future work includes recognizing an individual's brain states and decoding brain states from the fMRI data.

The work presented here provides proof‐of‐concept for the use of Geo‐Net4Net to identify distinct states that form dynamic functional networks based on tasks with known transition points. It was further demonstrated that using self‐supervised learning the information gained from the task‐based data can be applied to resting‐state periods. The HCP data used in this study only included a few short periods of rest (~20 s periods/scan). To demonstrate the application of the learned task transition point to the resting periods, it was assumed that the networks at rest were stationary. However, resting‐state dynamics have become of great interest in the neuroimaging field but doing group analyses of dynamic resting‐state networks poses a unique challenge. For a typical resting‐state scan (~5–10 min in duration), it has been found that the brain networks are not stationary and dynamic analyses can identify network topological changes over time. However, it can be challenging to assess state changes across participants because the brain states are not driven by a time‐controlled external condition like during a cognitive task. Thus, any given participant can be in any given state at any given time. Attempting to align these or identify state similarities across participants can be challenging. Hidden Markov modeling has been used to identify latent states in resting‐state fMRI for groups of participants (Vidaurre et al., 2017) has been used with success for groups of data. Our Geo‐Net4Net has the potential to be used to identify state transitions in groups and individual participants, a major focus of our future work.

Another direction of our future is to explore functional connectome biomarkers for diagnosing neurological diseases such as Autism and Alzheimer's disease. Indeed, it is straightforward to extend our proposed machine‐learning framework to disease diagnosis by replacing the current loss function (on change detection) with the softmax loss of classification on diagnosis labels.

## 5 CONCLUSIONS

We present a novel manifold‐based geometric neural network for functional brain networks (Geo‐Net4Net) to capture the intrinsic feature representations of FC, with the focus on the resting state. Specifically, we proposed to leverage the brain state change between tasks and resting state to guide the learning on the resting‐stage fMRI data that often does not have well‐defined ground truth. Since the geometry of functional brain networks is maintained in our DNN, we demonstrate a novel approach to characterize the functional fluctuations on the Riemannian manifold, where the well‐studied mathematical concepts allow us to visualize, quantify, and understand the resting‐state FC in the spectral domain. Our future work includes (1) downstream association between the low‐dimensional FC representations and the phenotype data and (2) developing a new fMRI simulation method based on the learned eigen‐spectrum.

## AUTHOR CONTRIBUTIONS

Guorong Wu and Paul J. Laurienti conceived the project, the main conceptual ideas, and the proof outline. Guorong Wu, Tingting Dan, and Zhuobin Huang developed the theory and performed the computations. Tingting Dan and Zhuobin Huang verified the analytical methods. Hongmin Cai encouraged Tingting Dan and Zhuobin Huang to investigate functional dynamics in resting state. Robert G. Lyday and Paul J. Laurienti analyzed and processed the data. Tingting Dan, Paul J. Laurienti, and Guorong Wu wrote the manuscript. All authors discussed the results.

## CONFLICT OF INTEREST

The authors have no conflicts of interest, financial or otherwise.

## Figures and Tables

**FIGURE 10:**
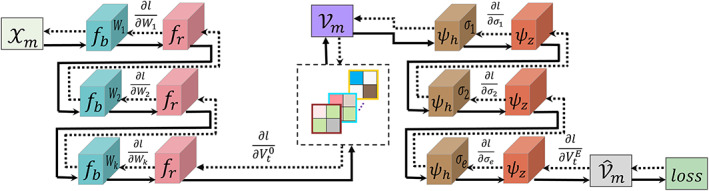
The feed‐forward (solid line) and back‐propagation (dashed line) flows in our Geo‐Net4Net

## Data Availability

The SimTB toolbox was utilized to generate the simulated fMRI time series. The toolbox is available in https://trendscenter.org/software/simtb/. The raw real data are collected from HCP (Human Connectome Project) database. The data that support the findings of our work are openly available in https://www.humanconnectome.org/.

## Footnotes

^1^
A pretext task is used in self‐supervised learning to generate useful feature representations that should be easily adaptable for other tasks (Jing & Tian, 2020).

## APPENDIX A ESTIMATE THE FRÉCHET MEAN FROM A SET OF SPD MATRICES ON THE RIEMANNIAN MANIFOLD

### A.1

Suppose X is the SPD matrix, that is, X∈M. The log operation is used to project X to the tangent plane $T_{R}M$ at R (R∈M) by $y=\log_{R}X=R^{\frac{1}{2}}\log\left(R^{-\frac{1}{2}}XR^{-\frac{1}{2}}\right)R^{\frac{1}{2}}$. On the contrary, the exp operation is used to project a vector $y\in T_{R}M$ back to the manifold M by $X=\exp_{R}\left(y\right)=R^{\frac{1}{2}}\exp\left(R^{-\frac{1}{2}}yR^{-\frac{1}{2}}\right)R^{\frac{1}{2}}$.

Given T SPD matrices $X_{t}$ (t=1,…,T), the Fréchet mean $\bar{X}$ can be iteratively estimated by repeating (1) project each $X_{t}$ to the tangent plane of the current subject‐specific mean $\bar{X}$ using log operation, denoted by $s_{t}=\log_{\bar{X}}X_{t}$, (2) calculate the mean tangent vector $s_{t}$ across time points using arithmetic averaging $\bar{s}=\sum_{t=1}^{T}s_{t}$, and (3) update the $\bar{X}$ by mapping the mean tangent vector back to the Riemannian manifold using the exp operation $\bar{X}=\exp_{\bar{X}}\left(\bar{s}\right)$. We repeat these three steps until converge.

## APPENDIX B MEAN‐SHIFT ON THE RIEMANNIAN MANIFOLD OF SPD MATRIX

### B.1

Suppose we have a series of the low‐dimensional geometric FC representations $V_{m}=\left\{V_{m}^{t}|t=1,\ldots ,T,{\;V}_{m}^{t}\in \mathrm{Sy}m_{P}^{+}\right\}\;$ for each subject. Mean‐shift simultaneously moves each instance $V_{m}^{t}$ toward its corresponding distribution mode and updates the latent mode from the new distribution of $V_{m}$. Since each $V_{m}^{t}$ is an SPD matrix, we tailor the iterative process of mean‐shift using the algebra on the Riemannian manifold, which consists of the following two steps.

1. Calculate the mean‐shift vector $Z_{m}^{t}=\psi_{h}\left(V_{m}^{t},\sigma \right)=\frac{\sum_{t\prime =1}^{T}\;h_{m}^{tt^{\prime }}\left[\log\left(V_{m}^{t\prime }\right)-\log\left(V_{m}^{t}\right)\right]}{\sum_{t\prime =1}^{T}\;h_{m}^{tt^{\prime }}}$, where $h_{m}^{tt^{\prime }}=\exp\left(-{\left[\frac{g_{m}^{\mathrm{tt}\prime }}{\sigma }\right]}^{2}\right)$ denotes Gaussian kernel, $g_{m}^{\mathrm{tt}\prime }$ is the geodesic distance between ${\;V}_{m}^{t}\;$ and $V_{m}^{t\prime }$, σ is the kernel width.
2. Update $V_{m}^{t}$ through mean shift by $V_{m}^{t}=\psi_{z}\left(V_{m}^{t},Z_{m}^{t}\right)=\exp\left(\log\left(V_{m}^{t}\right)+Z_{m}^{t}\right).$ Note that $\log\left(∙\right)$ and $\exp\left(∙\right)$ are Riemannian inverses for exponential and logarithmic mapping described by Subbarao and Meer (2009).

## APPENDIX C BACK‐PROPAGATION TO SOLVE THE NETWORK MINIMIZATION

### C.1

Our network architecture is provided by Figure 10. Since the loss function Equation (4) eventually examines the mis‐stratification based on each FC representation in ${\hat{V}}_{m}$, we take the *m*th subject as an example to illustrate the updating of network parameters in the back‐propagation process. For clarity, we abbreviate every FC matrix $V_{m}^{t}\;$ at *E*th iteration of MS‐RNN as $V_{t}^{E}$ (drop subscript m). The matrix logarithm of $V_{t}^{E}$ is given by $L_{t}^{E}=\log\left(V_{t}^{E}\right).$ Thus, the back‐propagation process starts from the gradient $\partial l/\partial V_{t}^{E^{\prime }}\;$ which can be derived from $\partial l/\partial L_{t}^{E}$ (Huang & Gool, 2017). $\frac{\partial l}{\partial L_{t}^{E}}=\sum_{t=1}^{T}\;\left(\frac{\partial l}{\partial g^{\mathrm{tt}\prime }}\frac{\partial g^{\mathrm{tt}\prime }}{\partial L_{t}^{E}}+\frac{\partial l}{\partial g^{t\prime t}}\frac{\partial g^{t\prime t}}{\partial L_{t}^{E}}\right),$ where $\frac{\partial g^{\mathrm{tt}\prime }}{\partial L_{t}^{E}}=\frac{L_{t}^{E}-L_{t\prime }^{E}}{{∥L_{t}^{E}-L_{t\prime }^{E}∥}_{F}},\frac{\partial l}{\partial G}=-\frac{\partial l}{\partial D}⊙\frac{1}{{\left(1_{T\times T}+G\right)}^{2}}$, $\frac{\partial l}{\partial D}=\frac{\partial l}{\partial \ell }⊙\left(1_{T\times T}-2B\right)$ and $\frac{\partial l}{\partial \ell }=\frac{1}{T^{2}}1_{T\times T}$, where B denotes binary indicator matrix, if $y_{m}^{t}=y_{m}^{t\prime }$, $B_{\mathrm{tt}\prime }=1$, otherwise, $B_{\mathrm{tt}\prime }=0,$ ⊙ denotes the element‐wise multiplication. Following the gradient chain in the study by Kong and Fowlkes (2018), we can derive the gradient $\frac{\partial l}{\partial V_{t}^{0}}$ at the beginning of MS‐RNN. As shown in Figure 10, the connection between SPD‐DNN and MS‐RNN is the FC representations $V_{t}^{0}$ for the *m*th subject. Given $\frac{\partial l}{\partial V_{t}^{0}}$, we follow the manifold‐based operations in the study by Huang and Gool (2017) to continue the back‐propagation in SPD‐DNN until we reach $\frac{\partial l}{\partial W_{1}}$. We use the stochastic gradient descent algorithm to optimize network hyperparameters ϕ and Θ sequentially.
